# Delayed Reconstruction of Palatomaxillary Defect Using Fibula Free Flap

**DOI:** 10.3390/jcm9030884

**Published:** 2020-03-24

**Authors:** Soo-Hwan Byun, Ho-Kyung Lim, Byoung-Eun Yang, Soung-Min Kim, Jong-Ho Lee

**Affiliations:** 1 Department of Oral & Maxillofacial Surgery, School of Dentistry, Seoul National University, Seoul 03080, Korea; purheit@daum.net (S.-H.B.); ungassi@naver.com (H.-K.L.); smin5@snu.ac.kr (S.-M.K.); 2 Department of Oral & Maxillofacial Surgery, Dentistry, Sacred Heart Hospital, Hallym University College of Medicine, Anyang 14068, Korea; face@hallym.or.kr; 3 Graduate School of Clinical Dentistry, Hallym University, Chuncheon 24252, Korea; 4 Department of Oral & Maxillofacial Surgery, Dentistry, Korea University Guro Hospital, Seoul 08308, Korea; 5 Oral Cancer Center & Clinical Trial Center, Seoul National University Dental Hospital, Seoul 03080, Korea; 6 Dental Research Institute, School of Dentistry, Seoul National University, Seoul 03080, Korea; 7 Clinical Translational Research Center for Dental Science, Seoul National University Dental Hospital, Seoul 03080, Korea

**Keywords:** reconstruction, palatomaxillary, defect, simulation, osteomyocutaneous flap, fibula free flap, maxilla, 3D printing, CAD/CAM, radiotherapy

## Abstract

Introduction. The objective of this study was to evaluate a surgical technique and to present the results of delayed reconstruction of palatomaxillary defects using fibula free flap (FFF). Methods. A review was conducted for nine patients who underwent palatomaxillary reconstruction using FFF. Primary disease, type of reconstruction, defect area, fibula segment length and number of osteotomies, radiotherapy, and implant installation after FFF reconstruction were analyzed. Results. All nine patients underwent delayed reconstruction. The fibula shaft was osteotomized into two segments in seven patients and three segments in one patient with bilateral Brown’s revised classification IV/d defect. One case was planned by using a computer-aided design computer-aided manufacturing (CAD/CAM) system with a navigation system. The mean length of the grafted fibula bone was 68.06 mm. Dental implant treatment was performed in six patients. Six patients received radiation therapy, and there were no specific complications related to the radiation therapy. In one case, the defect was reconstructed with FFF flow-through from a radial forearm free flap. Conclusion. This clinical study demonstrated that the fibula flap is an ideal donor-free flap in a palatomaxillary defect. Delayed reconstruction using an FFF can reduce the complication and failure rates.

## 1. Introduction

Oral and maxillofacial defects after surgical resection of malignant tumors frequently involve the maxilla with the palate. This kind of defect can also be a result of benign lesion or trauma and include the lining mucosa, teeth, and bone. Palatomaxillary defects sometimes extend over the midline and, if not adequately reconstructed, can result in poor esthetic and functional outcomes. The defects are problematic not only for the patients in functional and psychological aspects but also for the surgeons who try to achieve the ideal outcomes. Restoration of these defects would be a great challenge for clinicians.

The maxilla is a multifunctional bony structure that provides mechanical support to the midfacial area and contributes to esthetic appearance. It provides mechanical support to the orbit and bony framework for the maxillary dentition [1]. In addition, maxilla is an essential structure for functions including swallowing, speech, and articulation which are dependent on the integrity of the palatal and alveolar arch [1]. Failure to rehabilitate such functions when composing an entire reconstructive planning may result in significant quality of life issues [1].

There are several methods to restore palatal and maxillary defects [2,3,4]. Previous studies comparing the obturator and microvascular free flap reconstruction techniques have reported variable results. Breeze et al. compared the health-related quality of life in patients who underwent rehabilitation with obturators and reconstruction with a flap [5]. There were no specific differences between the obturator rehabilitation and flap reconstruction techniques, regardless of the size of the vertical defect or whether patients received radiation therapy [5]. In contrast, other studies have presented improved patient’s satisfaction, speech, and swallowing after flap reconstruction of large palatomaxillary defects compared to obturator rehabilitation [6,7].

A considerable number of surgical techniques has been used to reconstruct palatomaxillary defects. In previous studies, contouring of various soft tissue and bony free flaps including fibula, rectus abdominis, scapula, anterolateral thigh, radial forearm, and latissimus dorsi gained popularity for maxillary reconstruction [8]. All of these free flaps have been preferred for their advantages and disadvantages [9]. Flaps most commonly used for maxillofacial reconstruction are the fibula free flap, scapula free flap, and the iliac crest free flap [8].

Reconstruction of maxillofacial defects with the fibula free flap is a well-established technique [10,11]. Fibula provides a sufficient bone that can be designed to reconstruct various kinds of defect. The fibula free flap also has the advantages of a soft skin paddle, multiple osteotomies, a long pedicle, and proper bone shape for implant placement.

Studies have shown excellent outcomes of dental implant placement in the fibula after reconstruction with the fibula free flap [12,13,14,15]. The fibula also supports dental implants, and a skin paddle of the fibula free flap can be used with the bony component to provide the intraoral lining [11,16]. A skin paddle has been reported to be more mobile than attached gingiva, and the mobile skin paddle may predispose to poor oral hygiene [17]. A skin paddle is usually bulky and requires reduction in volume before placement in the defect to facilitate dental implant installation and to ensure normal speech.

The purpose of this study was to evaluate the outcomes of palatomaxillary defect reconstruction using the fibula free flap. The specific aim of the study was to show that three-dimensional model simulation and preoperative planning is an effective method to refine the procedure, to shorten the surgical time, and to predict postsurgical outcomes.

## 2. Materials and Methods

All of the records were reviewed for the patients who underwent reconstruction of midfacial defects treated in the maxillofacial surgery department of Seoul National University Dental Hospital. Institutional review board approval was obtained for this review (IRB 081/07-14). An author performed a retrospective review of medical record in all patients who received the reconstruction of midfacial defects. Nine consecutive cases of the fibula free flap with maxillofacial reconstruction were reviewed.

The 3D-printed model was manufactured in all of cases for cosmetic and functional reconstruction procedure in order to restore supportive function of midfacial structures and to apply the dental implants and further prosthetic rehabilitation. This procedure was performed in the following steps: analysis of the patient’s CT DICOM data; simulation of the obtained data into a three-dimensional (3D) image; and printing of the 3D model including the defect area with 3D printer [18]. The 3D model was utilized to plan and design the osteotomy of bone segments properly to the planned reconstruction. The 3D model of the bone segment was adequately shaped with polyacrylic resin (pre-shaped resin segment), considering the adjacent anatomic structures, and placed in the defect area of the 3D model before the surgery. Bony segment of the free flap was harvested and shaped according to the pre-shaped resin segment.

One case was planned by computer-aided design/computer aided manufacturing (CAD/CAM) system with navigation system (BrainLab image guided surgery, BrainLab^®^, Munich, Germany), which facilitated fabrication of the customized device to provide the patient with midfacial reconstruction. DICOM data of CT was imported into digital planning program (iPlan^®^ CMF, BrainLab^®^, Munich, Germany) and aligned during the planning process. The alignment of the dataset allowed a comparison between both sides of the patient in one view. The segmentation and mirroring of objects was the most time-consuming step when using digital planning software, where the objects were segmented accurately within 3 h. The mirrored data could be manipulated and positioned according to given anatomical structures of the affected side. The STL export system of the program allowed objects and anatomical structures to be exported for the production of patient-specific osteotomy guide or rapid prototyping models. The pre-shaped segment and osteotomy guide were manufactured using a 3D printing system. The fibula bone was designed and cut using the osteotomy guide during operation, and the navigation system was used to enhance the accuracy of placement of the fibular graft [19]. The results of digital planning could be improved by the acquisition of intraoperative images as well as fusion with preoperative digital planning data by using navigation system. 

**Figure 1:**
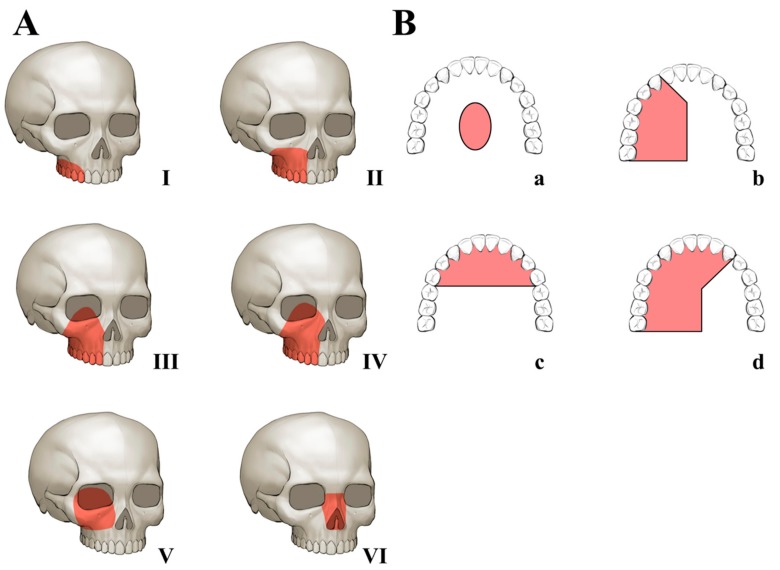
Brown’s revised classification of maxillary and midfacial defects [4]: (**A**) Vertical classification: I. maxillectomy not causing an oronasal fistula; II. not involving the orbit; III. involving the orbital area with orbital retention; IV. with orbital exenteration; V. orbitomaxillary defect; and VI. nasomaxillary defect. (**B**) Horizontal classification: a. palatal defect only, not involving the dental alveolus; b. less than or equal to 1/2 unilateral; c. less than or equal to 1/2 bilateral or transverse anterior; and d. greater than 1/2 maxillectomy.

All clinical data were analyzed, including primary disease, type of surgery, defect area, fibular bone length, number of osteotomies, and radiotherapy. Primary disease was confirmed by pathologic examination, and the defect area was classified according to the Brown’s revised classification of maxillary and midfacial defect [4] (Figure 1). A clinician measured the length of the grafted fibular bone and number of osteotomies using the postoperative CT and 3D-printed models.

## 3. Results

Nine patients met the inclusion criteria, and the patients lost to follow-up were excluded. Their ages ranged from 22 to 68 years, composed of 7 males and 2 females (Table 1). The previous operation history of resection was maxillectomy in 4 patients, palatal rotation flap with open reduction and internal fixation in 1 patient, maxillectomy with radial forearm free flap in 3 patients, and maxillectomy with rectus abdominis free flap in 1 patient before reconstruction. Follow-up period ranged from 36 to 235 months, with the mean of 121 months. The mean period was 99.33 months from resection to reconstruction in 8 patients. Delayed reconstruction was performed for sufficient oncologic monitoring after resection surgery. The composite defect of 1 patient was caused by a traffic accident; there were severe soft tissue defects in the maxillofacial area and multiple fractures of other parts. This patient underwent reconstruction using a local flap and received orthopedic treatment prior to the delayed reconstruction. All nine patients underwent delayed reconstruction to reduce the possibility of complications and recurrence.

Histologic causes of defect were squamous cell carcinoma in 2 patient, mucoepidermoid carcinoma in 3 patient, adenoid cystic carcinoma in 2 patients, and unknown cancer in 1 patient. One patient with unknown cancer was referred from another institution for reconstruction. It has been more than ten years since the patient underwent the resection surgery, so the histologic results of resection surgery were absent. All defects were graded as follows according to Brown’s revised classification of maxillary and midfacial defects: class II, III, or IV/b or d (IIb:1, IIId:1, IVb:1, and IVd:3). 

**Figure 2:**
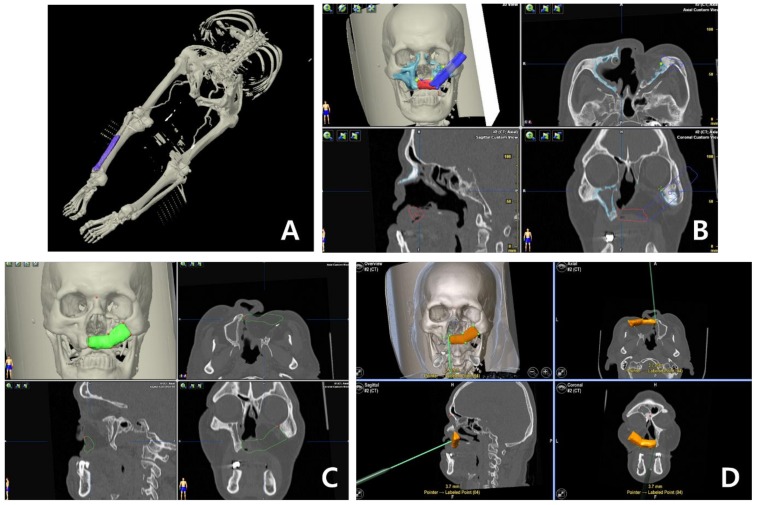
Preoperative procedure involving computer-aided design/computer-aided manufacturing (CAD/CAM) system with navigation system (BrainLab^®^) in order to enhance the accuracy and to reduce the operation time: (**A**) Lower extremity angio CT, (**B**) image export to the CAD program (BrainLab^®^), (**C**) the fibular bone is adjusted to the mirror image of contralateral side, and (**D**) navigation guide for the intraoperative procedure.

**Figure 3:**
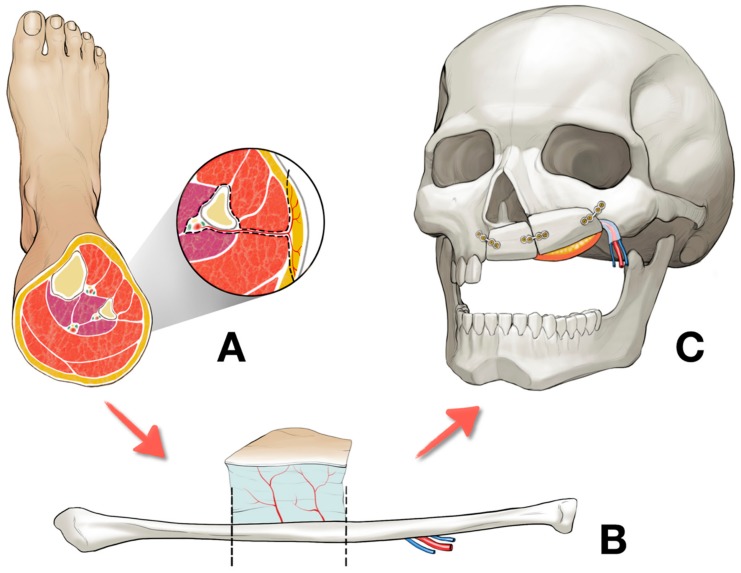
The entire procedure of the left palatomaxillary defect reconstruction using right fibula free flap: (**A**) Harvesting the right fibula free flap, (**B**) osteotomized into two segments, and (**C**) placement of the fibula free flap at the left palatomaxillary area.

**Figure 4:**
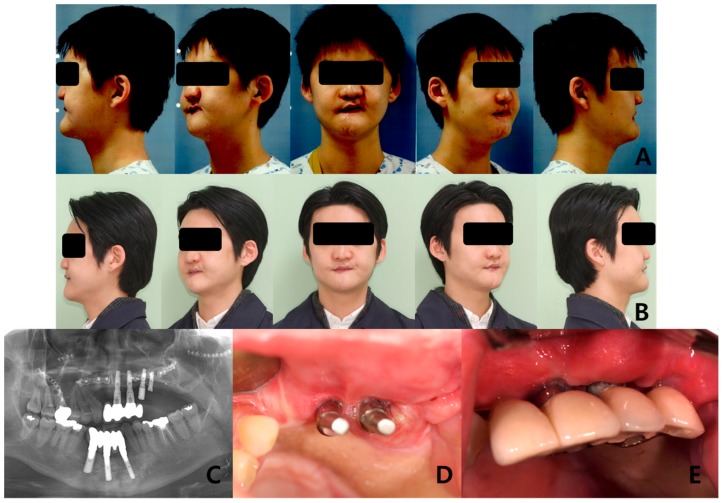
Long-term follow-up of the patients who received prosthetic rehabilitation with dental implant after the fibula free flap: (**A**) Preoperative clinical photos, (**B**) postoperative clinical photos after 4 years, (**C**) panoramic radiograph after implant treatment, (**D**) connection of the customized abutment, and (**E**) final prosthesis.

The fibular bone was osteotomized into two segments in seven patients and three segments in one patient with bilateral Brown’s revised classification IV/d defect. The defects on the midfacial area were on the right side in 4 patients and on the left side in 5 patients. At surgery, the mean length of fibular bone was 68.06 mm (Table 2). Of all the patients, 8 patients had only fibula free flap and 1 patient had fibula osteomyocutaneous flap flow-through from radial forearm free flap. In one case, the navigation surgery system (BrainLab^®^, Munich, Germany) was utilized with a special template technique and showed promising results for computer aided design/computer aided manufacturing (CAD/CAM) applications in reconstructive surgery (Figure 2). Of the 9 patients, 6 received postoperative radiation therapy, and reconstruction was carried out in the pre-irradiated area of these patients. Of the patients who received prosthetic rehabilitation, 6 received implant treatment while 3 had a prosthetic denture made to fit over their reconstructed skin flap site (Figure 3 and Figure 4). These implants were surrounded by healthy attached gingiva or nonkeratinized skin paddle. The overall success rate for 5 flaps was 100%.

## 4. Discussion

Maxillofacial trauma or tumor ablation often results in severe defects in the palatomaxillary area, and such defects require soft tissue, bone, and implant placement for the adequate restoration of structure and function [20]. Oral and maxillofacial reconstruction remains a significant challenge to surgeon.

The goals of reconstruction in palatomaxillary defect includes proper oronasal separation, provision of the alveolar arch, preservation of speech and occlusion, construction of structural framework of the midface, and provision of the midfacial projection while maintaining the nasal airway patency [21]. Debate remains on whether reconstruction is absolutely necessary in Brown’s revised classification class I or II. An obturator sometimes separates oral and nasoantral space adequately. However, most of the patients feel discomfort in swallowing and pronouncing. The patients are usually willing to reconstruct the midfacial defect, even if the outcomes of reconstruction would not fulfill their expectations. The reconstruction is absolutely essential, and the desire of the patients is important in a case of bilateral and enormous defect.

Fibula free flap was used for palatomaxillary reconstruction in this study. The use of the fibula free flap for palatomaxillary reconstruction has some advantages. The fibula bone provides 25 cm of bone in maximum for the reconstruction and the availability of shaping it. Its periosteal vascularization allows multiple osteotomies for esthetic reconstruction of palatomaxillary defect [22]. The bone structure of the fibula is very similar to the mandible, so this structural similarity provides a proper osteointegration of dental implants for prosthetic rehabilitation [22,23]. However, some clinicians have preferred using the angle of the scapula bone in maxillary reconstructions [24,25]. The decision-making of which free flap to choose for palatomaxillary reconstruction has often been based on individual preferences of the clinicians rather than on the scientific basis. Performing a flow-through subsequent free flap reconstruction would be more complicated than the single free flap reconstruction. The additional flow-through free flaps can be performed in patients with flap survival rates equivalent to those of initial free flaps [26]. If the midfacial defects include both the intraoral mucosa and extraoral skin, the surgeon should consider the additional flow-through free flap for coverage of intraoral mucosa or extraoral skin. Flow-through forearm free flap was used for the coverage of the paranasal skin defect in one case of this study.

In approximating fibula free flap to the defect, orientation of the vascular pedicle, the skin paddle, and the septal perforator must be considered in the preoperative procedure. In a cross-sectional view of the fibula free flap, the outline of fibular bone is triangular shape. The posterior intermuscular septum and the skin paddle are located at one of the angles. Septal perforators can be seen along a side between the angles, with the perforator coursing along the septum to the skin island [27]. The side related to the peroneus longus muscle is widest and flattest [27]. At surgery, the widest bone surface shows a favorable location to fix the fibular bone to the jaw with miniplates, with the vascular pedicle maintained behind the fibular bone to avoid the posterior intermuscular septum associated with the perforators [27]. Due to those reasons, the fibula free flap was harvested from the ipsilateral leg in this study.

The preoperative procedure involves the CAD/CAM system with navigation system in order to prepare device for assistance in the surgery [28,29]. The CAD/CAM system with a navigation system facilitated the fabrication of a customized device for virtual resection and reconstruction procedure out of the operation field [30,31,32]. Using this method including the navigation system which positioned the bony segment of fibula free flap to the exact position will reconstruct the resected area adequately [19,33]. The CAD/CAM system also provided data to manufacture a customized plate to reposition the grafted bone efficiently; however, since customized plates were not legal in Korea at that time, the fibula bone was to be fixed by a conventional plate.

Maxillofacial trauma or tumor ablation often results in compound defects in the maxillofacial region, and such defects require bone, soft tissue, and dental implants for the proper restoration of form and function [20]. The sufficient postoperative follow up must be needed to reduce the possibility of the further resection before the reconstruction. It seems reasonable to devote resources to restore function and aesthetics of the midfacial defect after the adequate postoperative follow up.

Dental implants would reconstruct the defects to improve the nutritional supply and psychological condition of patients after maxillofacial surgery. The implants are usually placed through the grafted skin paddle to restore function and esthetics after the reconstruction [34,35]. Byun et al. reported the implants placed through the skin paddle had a higher incidence of peri-implantitis (32.7%) than those placed through the mucosa (8.7%) [17]. Sclaroff et al. reported a success rate of 97.5% in a study on 83 dental implants in 16 patients with vascularized fibular flaps [36]. In the second patient who had been in a traffic accident, four implants were placed in the grafted fibula area; however, the two posterior implants showed peri-implantitis after connecting the prosthesis. Therefore, the prosthesis was modified only for the two anterior implants, while the posterior implants were submerged. Thick soft tissue and skin flap would have caused peri-implantitis. Implant placement was complicated in the palatomaxillary reconstruction, since the occlusion, soft tissue thickness, skin flap, and vascularization of the grafted fibula bone needed to be considered.

Six patients received radiation therapy. Free flap operation in patients who undergo radiotherapy is arduous as it involves dissecting easily nicked vessels and atrophied tissues. These factors are likely to lead to vascular damage and excessive bleeding during surgery. According to previous studies, free flap reconstruction of irradiated maxillofacial defects showed a lower success rate than that observed for non-irradiated defects [37,38,39]. In patients who have received radiotherapy at a dose over 60 Gy in the maxillofacial area, it is recommended to use recipient vessels outside the irradiated area to decrease the possibility of postoperative complications [39]. There were no specific complications in the irradiated patient in this study. There was a long-term monitoring period after radiotherapy, which would have reduced the radiation-induced fibrosis and endothelial injury [31,40]. If palatomaxillary reconstruction had been performed just after radiotherapy, the results of this study could have been different.

## 5. Conclusions

This clinical study demonstrates that fibula flap is an ideal donor-free flap in palatomaxillary reconstruction because of its length, thickness, and bone uniformity, which provide an ideal support for the adequate rehabilitation. The flow-through forearm radial flap not only serves as the vascular bridge to midface reconstruction but also provides sufficient soft tissue to cover the intraoral defect. The use of a CAD/CAM system and preoperative surgical planning is an effective method to refine reconstruction surgery, to enhance clinical outcomes, and to shorten operation time. Moreover, delayed reconstruction of the palatomaxillary defect using a fibula free flap can reduce complications and failure rate, especially in patients who have received radiotherapy.

## Figures and Tables

**Table 1:** Clinical data of enrolled patients with reconstruction of palatomaxillary defect.

Patient	Age	Cause of Defect	Previous Operation	Location of Defect
1	57	Adenoid Cystic Carcinoma	Maxillectomy with rectus abdominis free flap	Right maxilla
2	22	Traffic Accident	Palatal rotation flap	Left midface
3	64	Squamous Cell Carcinoma	Maxillectomy	Right maxilla
4	58	Adenoid Cystic Carcinoma	Maxillectomy	Left maxilla
5	63	Unknown cancer	Maxillectomy	Left midface
6	27	Mucoepidermoid Carcinoma	Maxillectomy	Left midface
7	38	Mucoepidermoid Carcinoma	Submaxillectomy with radial forearm free flap	Left maxilla
8	47	Mucoepidermoid Carcinoma	Partial maxillectomy with radial forearm free flap	Right maxilla
9	68	Squamous cell carcinoma	Hemimaxillectomy with radial forearm free flap	Right maxilla

**Table 2:** Clinical outcomes after the reconstruction of palatomaxillary defect.

Patient	From Resection to the Reconstruction	Bone Length (mm)	No. of Osteotomy	Skin	Number of Implants	Previous Radiotherapy
1	40M	66	1	Yes	3	Yes
2	TA *	66	1	Yes	4	No
3	19M	81	1	Yes	None	Yes
4	20M	120	2	Yes	4	Yes
5	136M	64.5	1	Yes	None	Yes
6	66M	57	1	Yes	3	No
7	74M	46	0	Yes	None	No
8	56M	45	1	Yes	3	Yes
9	36M	67	1	Yes	3	Yes

* TA. Traffic accident.
