# Correction: Byun, S.-H. et al. Delayed Reconstruction of Palatomaxillary Defect Using Fibula Free Flap. *J. Clin. Med.* 2020, *9*, 884

**DOI:** 10.3390/jcm9061712

**Published:** 2020-06-02

**Authors:** Soo-Hwan Byun, Ho-Kyung Lim, Byoung-Eun Yang, Soung-Min Kim, Jong-Ho Lee

**Affiliations:** 1 Department of Oral & Maxillofacial Surgery, School of Dentistry, Seoul National University, Seoul 03080, Korea; purheit@daum.net (S.-H.B.); ungassi@naver.com (H.-K.L.); smin5@snu.ac.kr (S.-M.K.); 2 Department of Oral & Maxillofacial Surgery, Dentistry, Sacred Heart Hospital, Hallym University College of Medicine, Anyang 14068, Korea; face@hallym.or.kr; 3 Graduate School of Clinical Dentistry, Hallym University, Chuncheon 24252, Korea; 4 Department of Oral & Maxillofacial Surgery, Dentistry, Korea University Guro Hospital, Seoul 08308, Korea; 5 Oral Cancer Center & Clinical Trial Center, Seoul National University Dental Hospital, Seoul 03080, Korea; 6 Dental Research Institute, School of Dentistry, Seoul National University, Seoul 03080, Korea; 7 Clinical Translational Research Center for Dental Science, Seoul National University Dental Hospital, Seoul 03080, Korea

The authors sincerely apologize for the imperfections made during the collection of data and wish to make the following correction to the previous paper [1].

## Notes

Should be replaced with:
